# Advances and Future Directions in the Clinical Utility of Food Addiction

**DOI:** 10.3390/nu13020708

**Published:** 2021-02-23

**Authors:** Stephanie E. Cassin, Sanjeev Sockalingam

**Affiliations:** 1 Department of Psychology, Ryerson University, 350 Victoria St, Toronto, ON M5B 2K3, Canada; 2 Department of Psychiatry, University of Toronto, Toronto, ON M5T 1R8, Canada; 3 Centre for Mental Health, University Health Network, Toronto, ON M5G 2C4, Canada; 4 Bariatric Surgery Program, University Health Network, Toronto, ON M5T 2S8, Canada; 5 Centre for Addiction and Mental Health, 1025 Queen Street West, B1 2nd Floor, Room 2300, Toronto, ON M6J 1H4, Canada

The body of research examining the validity of food addiction and eating addiction far exceeds the research examining their clinical utility. Although neither food addiction nor eating addiction are officially recognized diagnoses, many individuals self-identify as “food addicts” and/or exceed the cut-offs on measures of addictive-like eating. To be clinically useful, a diagnosis should inform the treatment plan and predict clinical outcomes. This special issue presents a collection of articles, contributed by renowned experts, researchers, and clinicians spanning different disciplines, that adds to the knowledge on the clinical utility of food addiction and eating addiction. The articles in this collection include reviews [1,2,3,4,5] as well as original research utilizing a variety of methodologies and study designs such as clinical trials [6,7,8], cross-sectional studies [9,10], and surveys [11].

Oliveria et al. [10] examined the characteristics of individuals seeking treatment for food addiction and found that they were very likely to present with comorbid diagnoses (83% of patients). On average, patients presented with 2 to 3 comorbid conditions, with anxiety and mood disorders being the most common. They also reported impairment in psychological, physical, and social functioning, and food addiction was a significant predictor of social impairment even when controlling for binge eating, depression, and anxiety severity.

In their international survey of health care professionals who potentially work with patients presenting with addictive eating behaviour (e.g., dietitians, psychologists), Burrows et al. [11] reported that the majority of respondents had been asked about addictive eating before (72%) and were interested or very interested in receiving training regarding addictive eating. They specifically reported a need for training in assessment/diagnosis (77%) and evidence-based treatments (81%). Therefore, knowledge translation of and training in food addiction assessment and treatment is needed to build capacity amongst healthcare providers.

Wiss & Brewerton [3] contributed a helpful guide to this special issue that can assist with the assessment of food addiction and differential diagnosis. Specifically, they described a comprehensive approach for assessing food addiction that takes into consideration factors such as dietary restraint and comorbid psychiatric disorders. By helping to distinguish food addiction from other forms of eating pathology, this approach aims to guide case formulation and treatment planning. Importantly, the authors concluded that “one size will not fit all in food addiction treatment” (p. 17).

A number of authors contributed papers to this special issue that emphasize the need for individual-level and societal-level interventions for food addiction. In a sample of post-operative bariatric surgery patients, Cassin et al. [6] found that those with food addiction reported greater binge eating characteristics and psychiatric distress relative to those without, and there was preliminary evidence that a brief telephone-based cognitive behavioural therapy intervention may lead to short-term improvement in food addiction symptoms. Two other studies examined food addiction in the context of behavioural weight loss programs. In a sample of individuals with obesity and binge eating disorder participating in a behavioural weight loss program, Wiedemann et al. [7] found that those with food addiction reported a stronger negative reaction to weekly weighing and less acceptance of their weight and shape throughout treatment, and the authors recommended that body image concerns be targeted in treatment given that both of these factors prospectively predicted greater eating disorder psychopathology. Gordon et al. [8] found that food addiction symptoms improved during a behavioural weight loss program; however, more severe food addiction symptomatology was associated with less weight loss. Interestingly, reduced intake of hyperpalatable foods during the program was associated with short-term improvements in food addiction symptoms but not with long-term improvements in food addiction symptoms or weight, suggesting that the association among hyperpalatable foods, food addiction, and weight is a complex one.

In his review and commentary, Lustig [1] provides a compelling argument that “personal intervention must be balanced with societal intervention” (p. 17) and presents evidence that added sugar, and by extension the category of ultraprocessed foods, meets the criteria deemed necessary and sufficient for public health regulatory policy. He then proposes a number of societal interventions, including public education, taxation, subsidies, and restricted access, that have been found effective in reducing the risk and impact of other public health issues. Wiss, Avena, & Gold [4] present a conceptual biopsychosocial model showing how early adversity, trauma, and stress may become biologically embedded and interact with psychological, social, and environmental factors to increase the risk of addiction, including food addiction. Following this model, they recommend a multilevel approach for reducing the risk and impact of food addiction that includes both individual and public health interventions.

Other authors examined the similarities between food addiction and other forms of addiction, or the presence of addictive-like eating in other clinical populations. Zawertailo et al. [5] conducted a narrative scoping review to examine commonalities between food addiction and tobacco use disorder and identified some shared biopsychosocial vulnerability factors (e.g., childhood adversity, attachment insecurity, dopaminergic neurocircuitry) and underlying mechanisms that may help to inform treatment options. They also included the results of a small pilot study examining food addiction among individuals seeking treatment for tobacco use disorder. The research conducted to date has primarily examined food addiction among individuals with other forms of addictions or eating disorders given their overlap, and Stogios et al. [2] extended this line of research into a new clinical population in their scoping review of eating behaviours among individuals with psychosis. 

Collectively, the articles included in this special issue suggest that individuals with food addiction, and particularly those presenting for treatment of food addiction, often have comorbid psychiatric diagnoses, body image concerns, and impaired quality of life, and that many health care professionals who potentially work with such patients feel that they need additional knowledge of, and training in, assessment and evidence-based treatments for food addiction. Similar to other addictions such as tobacco use disorder, a multi-component approach including both individual and societal intervention is warranted to reduce the personal and public health impact of food addiction.

As research attention shifts from examining the validity to the clinical utility of food and eating addiction, many questions remain to be answered. What factors predict treatment seeking among individuals with food or eating addiction? What are the treatment preferences of individuals with food or eating addiction? Are existing evidence-based treatments for eating disorders or substance-related and addictive disorders effective among individuals with food or eating addiction? What can be done to improve the durability of treatment effects of those interventions that have already been examined and found to improve only short-term outcomes? What is the evidence for abstinence-based versus moderation approaches? How do patients experience each of these interventions? What do they find helpful and unhelpful, and what do they attribute any changes to? Recognizing that “one size will not fit all”, how can we move to personalized approaches to food addiction treatment (i.e., what treatment for whom)? We hope that the articles included in this special issue will provide the impetus to explore these important questions and generate knowledge to inform clinical practice guidelines.

## References

[1] Lustig R.H. (2020). Ultraprocessed Food: Addictive, Toxic, and Ready for Regulation. Nutrients.

[2] Stogios N., Smith E., Asgariroozbehani R., Hamel L., Gdanski A., Selby P., Sockalingam S., Graff-Guerrero A., Taylor V.H., Agarwal S.M. (2020). Exploring Patterns of Disturbed Eating in Psychosis: A Scoping Review. Nutrients.

[3] Wiss D., Brewerton T. (2020). Separating the Signal from the Noise: How Psychiatric Diagnoses Can Help Discern Food Addiction from Dietary Restraint. Nutrients.

[4] Wiss D.A., Avena N., Gold M. (2020). Food Addiction and Psychosocial Adversity: Biological Embedding, Contextual Factors, and Public Health Implications. Nutrients.

[5] Zawertailo L., Attwells S., deRuiter W.K., Le T.L., Dawson D., Selby P. (2020). Food Addiction and Tobacco Use Disorder: Common Liability and Shared Mechanisms. Nutrients.

[6] Cassin S., Leung S., Hawa R., Wnuk S., Jackson T., Sockalingam S. (2020). Food Addiction Is Associated with Binge Eating and Psychiatric Distress among Post-Operative Bariatric Surgery Patients and May Improve in Response to Cognitive Behavioural Therapy. Nutrients.

[7] Wiedemann A.A., Ivezaj V., Gueorguieva R., Potenza M.N., Grilo C.M. (2021). Examining Self-Weighing Behaviors and Associated Features and Treatment Outcomes in Patients with Binge-Eating Disorder and Obesity with and without Food Addiction. Nutrients.

[8] Gordon E.L., Merlo L.J., Durning P.E., Perri M.G. (2020). Longitudinal Changes in Food Addiction Symptoms and Body Weight among Adults in A Behavioral Weight-Loss Program. Nutrients.

[9] Jezewska-Zychowicz M., Małachowska A., Plichta M. (2020). Does Eating Addiction Favor A More Varied Diet or Contribute to Obesity?—The Case of Polish Adults. Nutrients.

[10] Oliveira E., Kim H.S., Lacroix E., de Fátima Vasques M., Durante C.R., Pereira D., Cabral J.R., Bernstein P.S., Garcia X., Ritchie E.V. (2020). The Clinical Utility of Food Addiction: Characteristics and Psychosocial Impairments in A Treatment-Seeking Sample. Nutrients.

[11] Burrows T., Verdejo-Garcia A., Carter A., Brown R.M., Andrews Z.B., Dayas C.V., Hardman C.A., Loxton N., Sumithran P., Whatnall M. (2020). Health Professionals’ and Health Professional Trainees’ Views on Addictive Eating Behaviours: A Cross-Sectional Survey. Nutrients.

